# Depression and Anxiety in Mothers Who Were Pregnant During the COVID-19 Outbreak in Northern Italy: The Role of Pandemic-Related Emotional Stress and Perceived Social Support

**DOI:** 10.3389/fpsyt.2021.716488

**Published:** 2021-09-03

**Authors:** Serena Grumi, Livio Provenzi, Patrizia Accorsi, Giacomo Biasucci, Anna Cavallini, Lidia Decembrino, Rossana Falcone, Elisa Maria Fazzi, Barbara Gardella, Roberta Giacchero, Paola Guerini, Elena Grossi, Maria Luisa Magnani, Eloisa Maria Mariani, Renata Nacinovich, Dario Pantaleo, Camilla Pisoni, Federico Prefumo, Caterina Sabatini, Barbara Scelsa, Maria Valentina Spartà, Arsenio Spinillo, Roberto Giorda, Simona Orcesi, Renato Borgatti

**Affiliations:** ^1^Child Neurology and Psychiatry Unit, IRCCS Mondino Foundation, Pavia, Italy; ^2^Unit of Child and Adolescence Neuropsychiatry, ASST Spedali Civili, Brescia, Italy; ^3^Pediatrics & Neonatology Unit, Guglielmo da Saliceto Hospital, Piacenza, Italy; ^4^Child and Adolescent Mental Health, San Gerardo Hospital, Monza, Italy; ^5^Pediatric Unit and Neonatal Unit, ASST Pavia, Pavia, Italy; ^6^Department of Clinical and Experimental Sciences, University of Brescia, Brescia, Italy; ^7^Department of Obstetrics and Gynecology, Fondazione IRCCS Policlinico San Matteo, Pavia, Italy; ^8^Department of Brain and Behavioral Sciences, University of Pavia, Pavia, Italy; ^9^Department of Pediatrics, ASST Lodi, Lodi, Italy; ^10^School of Medicine and Surgery and Milan Center for Neuroscience, Università Bicocca, Milan, Italy; ^11^Neonatal Intensive Care Unit, Fondazione IRCCS Policlinico San Matteo, Pavia, Italy; ^12^Pediatric Neurology Unit, V. Buzzi Children's Hospital, Milan, Italy; ^13^Biology Lab, Scientific Institute IRCCS E. Medea, Bosisio Parini, Italy

**Keywords:** COVID- 19, mothers, social support, stress, anxiety, depression, pandemic, pregnancy

## Abstract

The COVID-19 pandemic is a collective trauma that is threatening citizens' mental health resulting in increased emotional stress, reduced social support, and heightened risk for affective symptoms. The present study aimed to investigate the effects of antenatal pandemic-related emotional stress and perceived social support on the symptoms of depression and anxiety of mothers who were pregnant during the initial COVID-19 outbreak in northern Italy. A sample of 281 mothers was enrolled at eight maternity units in the first hotspot region of the COVID-19 outbreak in northern Italy. Participants filled out online questionnaires assessing the direct or indirect exposure to the SARS-CoV-2 virus, pandemic-related stress, perceived social support, as well as symptoms of depression and anxiety. Depressive and anxious symptomatology was above clinical concern, respectively, in 26 and 32% of the respondents. Mothers who reported no exposure to SARS-CoV-2 during pregnancy and those who reported at least one direct or indirect exposure did not differ in terms of affective symptoms. Continuous scores and risk for severe depression and anxiety were positively associated with prenatal pandemic-related emotional stress and negatively linked with perceived social support during pregnancy. Women who become mothers during the COVID-19 emergency may be at high risk for affective problems. Dedicated preventive programs are needed to provide adequate preventive support and care for maternal mental health during and after the COVID-19 pandemic.

## Introduction

The coronavirus disease of 2019 (COVID-19) has rapidly spread worldwide during the first months of 2020 and it is now acknowledged as an unprecedented pandemic (1). Among European countries, Italy was dramatically hit, and the northern area of the country was the first region to be locked down to contain and mitigate the contagion (2). The SARS-CoV-2 was confirmed to be spreading in Italy on January, 31st 2020 and the contagion followed an exponential trend, leading to more than ten thousand confirmed infected patients and more than 800 deaths on March, 11th 2020 (*ibidem*). As we are writing in July 2021, more than 4 million Italian people have been infected and deaths with COVID-19 are more than 120,000.^1^ The high risk of COVID-19 infection—together with the lack of clear scientific knowledge about the SARS-CoV-2 virus—represented a direct (e.g., risk of contagion) and indirect (e.g., worries for significant others' contagion and socio-economic impact) risk factor for citizens' mental health (3, 4). The psychological and stressful consequences of the COVID-19 emergency should not be underestimated in fragile individuals and during specific sensitive developmental windows, such as pregnancy and neonatal life (5).

Rapidly accumulating research is suggesting that women may not be at higher risk for severe COVID-19 illness during pregnancy and in the postnatal period (6–8). Nonetheless, the pandemic is a collective traumatic experience that may indirectly affect the mental health of expecting women and mothers increasing the levels of perceived stress during a period of heightened plasticity (9, 10). There is extensive proof that prenatal stress may pave the way to post-natal symptoms of depression and anxiety (11–15) that may later develop into full-blown affective disorders (16, 17). Not surprisingly, studies conducted during the first months of the COVID-19 healthcare emergency are highlighting high levels of stress and reduced psychosocial well-being among pregnant women and mothers during the pandemic (18, 19). A meta-analytic study reported that levels of depression were higher during the present pandemic when compared to previous reports during non-pandemic times (20). Nonetheless, greater risk has been documented for symptoms of anxiety, which were among the most reported psychological symptoms in pregnant women and mothers in different countries hit by the COVID-19 pandemic (21–24).

Although mild elevations in depressive and anxious symptomatology may be observed after delivery in healthy and low-risk samples, it should be highlighted that identifying and targeting these symptoms appropriately may be key to the success of preventive interventions (25). Exposure to antenatal maternal stress predicts a wide variety of behavioral, emotional, cognitive, and physical outcomes in the offspring (26, 27). Maternal stress experienced during pregnancy may negatively impact temperamental development (28), attentional processes (29), and stress regulation (30) during infancy and childhood (31, 32).

Notably, the maternal perception of social support may be a source of significant buffering in the face of prenatal stress and adverse psychological conditions during pregnancy, contributing to reduce the risk of affective symptoms postnatally (33–35). As an indirect side effect of mitigation and containment strategies, women who were pregnant during the COVID-19 pandemic may have experienced reduced social support during pregnancy and this may have in turn contributed to further elevate their levels of emotional stress (22, 36). Previous research has largely documented that perceived social support during pregnancy may be beneficial for the short- and long-term mental health of mothers. In a large longitudinal cohort, greater maternal perceived social support predicted lower stress and anxiety (37), and these findings have been replicated even in samples of women exposed to collective traumas [e.g., the Iowa flood study; (38)]. Despite the literature on maternal mental health has rapidly grown during the first months of the COVID-19 emergency, less is known for what pertains to the effects of social support experienced by pregnant women on subsequent symptoms of depression and anxiety.

### The Goals of the Present Study

The primary goal of the present study was to assess the presence of a statistically significant difference in depressive and anxious symptoms among mothers who reported at least one direct or indirect exposure to the SARS-CoV-2 virus and those who reported no such exposures. We hypothesized that mothers who had greater exposure to the SARS-CoV-2 had higher levels of affective symptoms. A second goal was to assess the presence of a statistically significant association between prenatal emotional stress response to the pandemic and both depressive and anxious symptoms after delivery. Based on previous research (11–17), we hypothesized that mothers who reported higher levels of stress before delivery also had higher levels of postnatal depression and anxiety. Finally, a third goal was to investigate the presence of a statistically significant association between social support during pregnancy and postnatal symptoms of depression and anxiety. According to the social support literature reported above (33–35, 37), we hypothesized that mothers who experienced higher social support during pregnancy had lower levels of anxiety and depression postnatally.

## Method

### Participants and Procedures

This study is part of the longitudinal and multi-centric research project entitled Measuring the Outcomes of Maternal Covid-19-related Prenatal Exposure (MOM-COPE) (25). In the present manuscript, we report on a sample of 281 mothers. Participating women were enrolled between May 15th and December 28th, 2020 from eight hospitals geographically located within the first hotspot of the Italian COVID-19 outbreak. Mothers were included in the MOM-COPE project if at least 18-year-old, in absence of prenatal and perinatal risk factors, if they delivered at term (i.e., from 37^+0^ to 41^+6^ weeks of gestation), cohabiting with the infant's father, and if they were negative for COVID-19 at delivery. Mothers were first contacted at antepartum classes or immediately following the postpartum period. Socio-demographic and neonatal data were obtained from medical records. Within 48 h of delivery, mothers were asked to fill in questionnaires through an online digital platform (see below). The study was approved by the Ethics Committees of the project lead institution (IRCCS Mondino Foundation, Pavia, Italy) and the participating hospitals. All mothers provided informed consent to participate in the study.

### Measures

The exposure to the SARS-CoV-2 virus was assessed using seven dichotomous items (0, no; 1, yes) targeting direct (e.g., “I was infected during pregnancy”) and indirect (e.g., “One of my friends or relatives died from COVID-19”) exposures. A global exposure score was obtained by summing these items and participants were grouped into those exposed to SARS-CoV-2 (exposure >1, exposed subjects) and those with no direct nor indirect exposure (exposure = 0, non-exposed subjects). The pandemic-related emotional stress response to the COVID-19 emergency during pregnancy was assessed with six 5-point Likert scale items (1, not at all; 5, very much) (25). An average emotional stress score was obtained by averaging the score of all the emotional stress items. The internal consistency for the emotional stress questionnaire was satisfactory (Cronbach's α = 0.84). The items related to COVID-19 exposure and emotional stress are reported in Table 1.

**Table 1:** *Ad-hoc* questionnaires to assess exposure to SARS-CoV-2 and emotional stress response to the healthcare emergency.

**A**.	**Exposure to SARS-CoV-2 (Response: Yes, No)**
	**During pregnancy…**
1	I tested positive for COVID-19
2	I had symptoms reminiscent of COVID-19
3	I had contacts with relatives or friends who tested positive for COVID-19
4	I live in a high contagion zone (e.g., red zone)
5	I had contacts with relatives or friends who live in a high contagion zone (e.g., red zone)
6	One of my relatives or friends was hospitalized due to the COVID-19 infection
7	One of my relatives or friends died with COVID-19
**B**.	**Emotional stress (Response: 5-point Liker scale, 1=** **not at all;** **2=** **slightly; 3=** **Moderately; 4=** **Very much; 5=** **Extremely)**
	**During pregnancy…**
1	How much worried were you about the risk of COVID-19 infection?
2	How much did you feel that your pregnancy was at risk due to the COVID-19 pandemic?
3	How much did you fear for your health?
4	How much did you fear for your baby's health?
5	How much did you feel that you were losing confidence in your health?
6	How much did you feel you had lost faith in medicine?

Perceived social support during pregnancy was assessed using the Italian version (39) of the Multidimensional Scale of Perceived Social Support [MSPSS; (40)]. The MSPSS consists of 12 items and assesses perceptions of support from three different sources: family, friends, and significant others. The MSPSS global score (range 12–84) was used in the present study to estimate the perceived social support experienced by the women during pregnancy. Symptoms of depression and anxiety were assessed within 48 h using the Beck Depression Inventory [BDI-II; (41)] and the State-Trait Anxiety Inventory [STAI-Y; (42)], respectively. The Italian version of the BDI-II (43) is a 21-item self-report questionnaire that provides a descriptive and non-diagnostic account of the severity of symptoms of depression. Each item is rated on a 4-point Likert scale and the total continuous score ranges from 0 (low) to 63 (high). BDI-II scores lower than 13 indicate a low risk of severe depression, whereas scores of 13 or above are indicative of a high risk for severe symptoms. The state anxiety subscale of the Italian version of the STAI-Y (44) features twenty 4-point Likert-scale items and provides a descriptive and non-diagnostic account of the severity of symptoms of anxiety. The total continuous score ranges from 20 (low) to 80 (high). Scores lower than 40 suggest a low risk of severe anxiety, whereas scores equal to or higher than 40 are reminiscent of elevated risk of anxious symptomatology.

### Plan of Analysis

Exposed and non-exposed participants were compared for pandemic-related emotional stress, depressive symptoms, social support, and anxious symptoms using independent-sample *t*-tests (Goal 1). To assess the association between prenatal pandemic-related emotional stress and both symptoms of depression and anxiety (Goal 2), separate Spearman bivariate correlation coefficients were computed using the continuous BDI-II and STAI-Y score. Moreover, to further assess the role of pandemic-related emotional stress in increasing the risk of depressive and anxious symptomatology, binary logistic regressions were used to estimate the effect of prenatal emotional stress related to the pandemic on the dichotomous BDI-II and STAI-Y scores. To assess the association between prenatal perceived social support and both symptoms of depression and anxiety (Goal 3), separate Spearman bivariate correlation coefficients were computed using the continuous BDI-II and STAI-Y score. Additionally, to further assess the role of social support in decreasing the risk of depressive and anxious symptomatology, binary logistic regressions were used to estimate the effect of prenatal emotional stress related to the pandemic on the dichotomous BDI-II and STAI-Y scores. Statistical analyses were conducted using SPSS 27 for Windows setting *p* < 0.01.

## Results

Descriptive statistics are reported in Table 2 for the whole sample as well as separately for COVID-19 exposed and non-exposed women. Generally, 167 (59.4%) mothers were exposed—directly or indirectly—to the virus during pregnancy, whereas 114 (40.6%) reported no exposure. Only one mother was positive for COVID-19, less than half of them (*n* = 114, 41%) reported no physical direct or indirect exposure to the SARS-CoV-2 virus, 74 (26%) had a relative or close friend who was hospitalized for intensive care, and 40 (14%) experienced the death of a relative or close friend. Considering the whole sample, symptoms of depression and anxiety were above the clinical relevance cut-off in 72 (26%) and 90 (32%) mothers, respectively. Exposed and non-exposed mothers did not statistically differ in the distribution of the dichotomous BDI-II score [respectively: 45 (27.0%) and 27 (23.7%); χ^2^ = 0.38, *p* = 0.539] and STAI-Y score [respectively: 52 (31.1%) and 38 (33.3%); *X*^2^ = 0.15, *p* = 0.699].

**Table 2:** Descriptive statistics for the whole sample and subjects exposed or non-exposed to COVID-19 SARS-CoV-2.

		**All (** ***N*** **=** **281)**		**Exposure (** ***N*** **=** **167)**		**Non-exposure (** ***N*** **=** **114)**		***t***	***p***
		**Mean**	**SD**	**Mean**	**SD**	**Mean**	**SD**		
Gestational age (weeks)		39.72	1.04	39.69	1.04	39.76	1.03	0.21	0.83
Birth weight (grams)		3,358.56	424.16	3,376.95	435.07	332.08	408.52	0.49	0.62
Head circumference (cm)		34.26	1.15	34.26	1.21	34.26	1.05	−0.01	0.99
Neonatal length (cm)		50.36	1.93	50.41	1.99	50.31	1.85	−0.43	0.67
Apgar (min 1)		9.18	0.68	9.17	0.68	9.18	0.68	0.49	0.63
Maternal age at delivery (years)		33.20	4.65	33.91	4.25	34.38	4.41	−0.74	0.46
Emotional stress		2.52	0.72	2.59	0.75	2.43	0.67	1.83	0.07
Social support		5.98	1.18	5.88	1.31	6.05	1.07	0.03	0.24
Depressive symptoms		6.31	5.65	6.39	5.73	6.20	5.57	0.71	0.79
Anxious symptoms		35.71	10.18	35.62	10.11	35.84	10.31	0.92	0.86
		***N***	**%**	***N***	**%**	***N***	**%**	***X**^**2**^*	***p***
Delivery	Vaginal	194	69.0	124	72.1	77	65.4	1.73	0.42
	Operative	20	7.1	10	6.5	7	6.5		
	Cesarean section	48	17.1	33	21.4	30	28.1		
Infants' sex	Females	138	49.1	82	49.4	56	49.5	0.01	0.92
	Males	143	50.9	85	50.6	58	50.5		
Maternal educational level	Primary school	23	8.2	12	7.2	11	9.6	2.01	0.57
	Secondary school	116	41.3	73	43.7	43	37.7		
	Bachelor/master	126	44.8	74	44.3	52	45.6		
	Post-graduate	16	5.7	8	4.8	9	7.0		
Exclusive breastfeeding	Yes	180	64.1	107	64.1	73	64.0	<0.01	0.99
	No	101	35.9	60	35.9	41	36.0		

### Depression and Anxiety Between Exposed and Non-exposed Mothers

**Figure 1:**
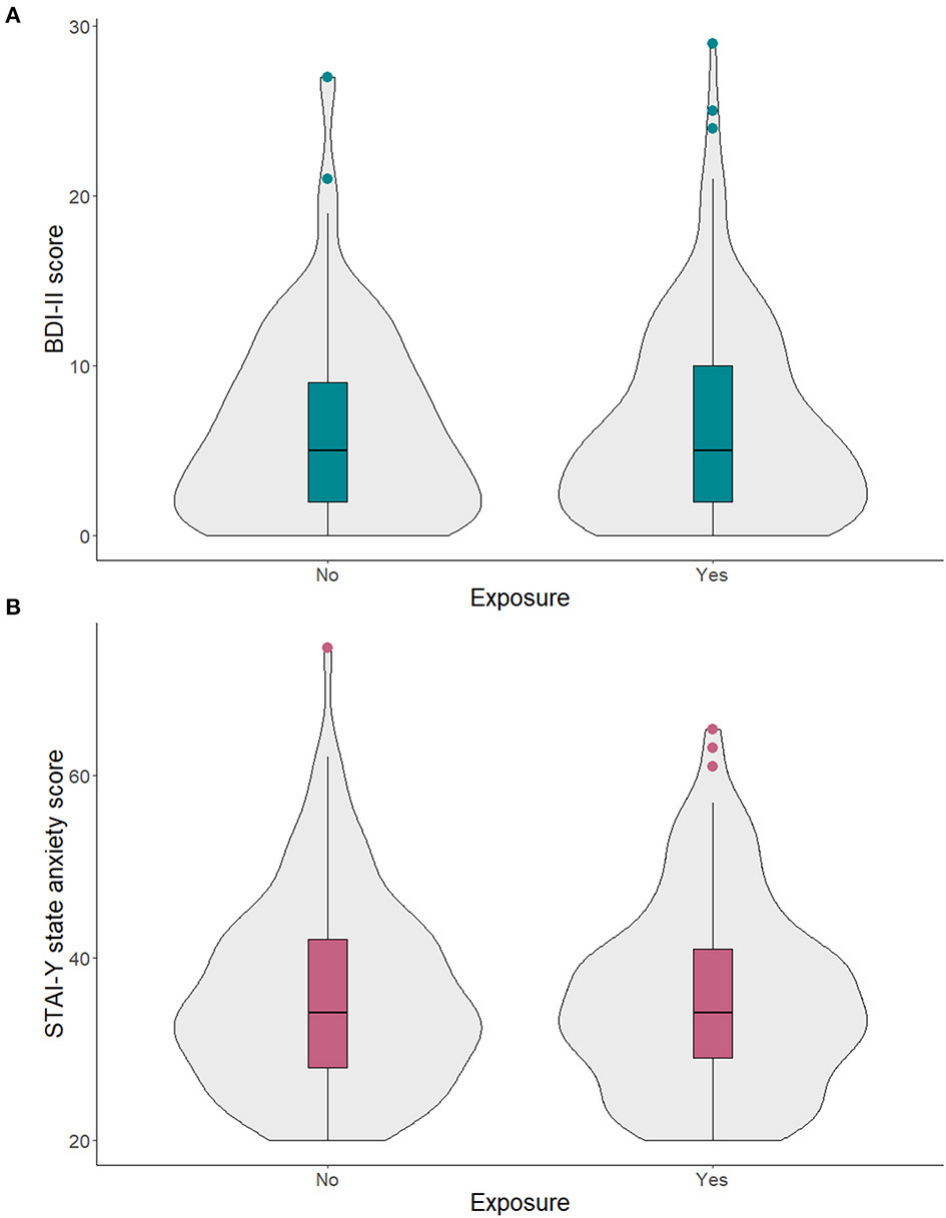
Continuous scores of symptoms of depression **(A)** and anxiety **(B)** in mothers who self-reported to have been exposed and non-exposed to the SARS-CoV-2.

Mothers who reported no exposure to the SARS-CoV-2 virus and those who reported at least one direct or indirect exposure did not differ in terms of emotional stress (Table 2). No significant differences between exposed and non-exposed mothers emerged for symptoms of depression and anxiety (Figure 1).

### Pandemic-Related Emotional Stress During Pregnancy and Maternal Mental Health

**Figure 2:**
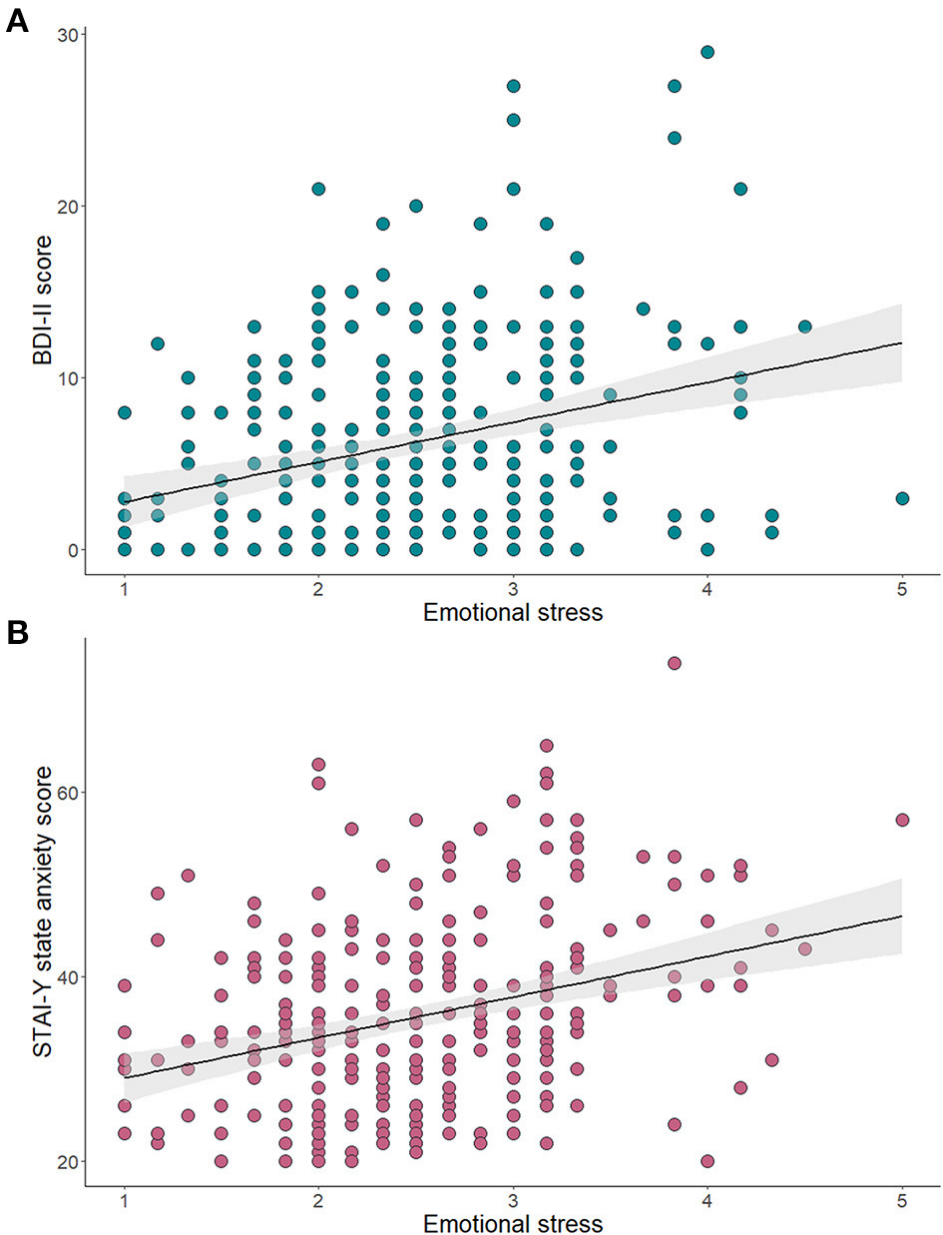
Association of pandemic-related emotional stress and continuous scores of symptoms of depression **(A)** and anxiety **(B)**.

Prenatal pandemic-related emotional stress was significantly and positively associated with both the BDI-II, *r* = 0.30, *p* < 0.001, and the STAI-Y, *r* = 0.31, *p* < 0.001 (Figure 2). One unit increase in emotional stress was significantly associated with a higher risk of developing clinically significant anxious, B = 0.80, *p* < 0.001, Exp(B) = 2.23, 95% C.I. (1.51:3.28), and depression, B = 0.89, *p* < 0.001, Exp(B) = 2.44, 95% C.I. (1.61:3.69).

### Perceived Social Support During Pregnancy and Maternal Mental Health

**Figure 3:**
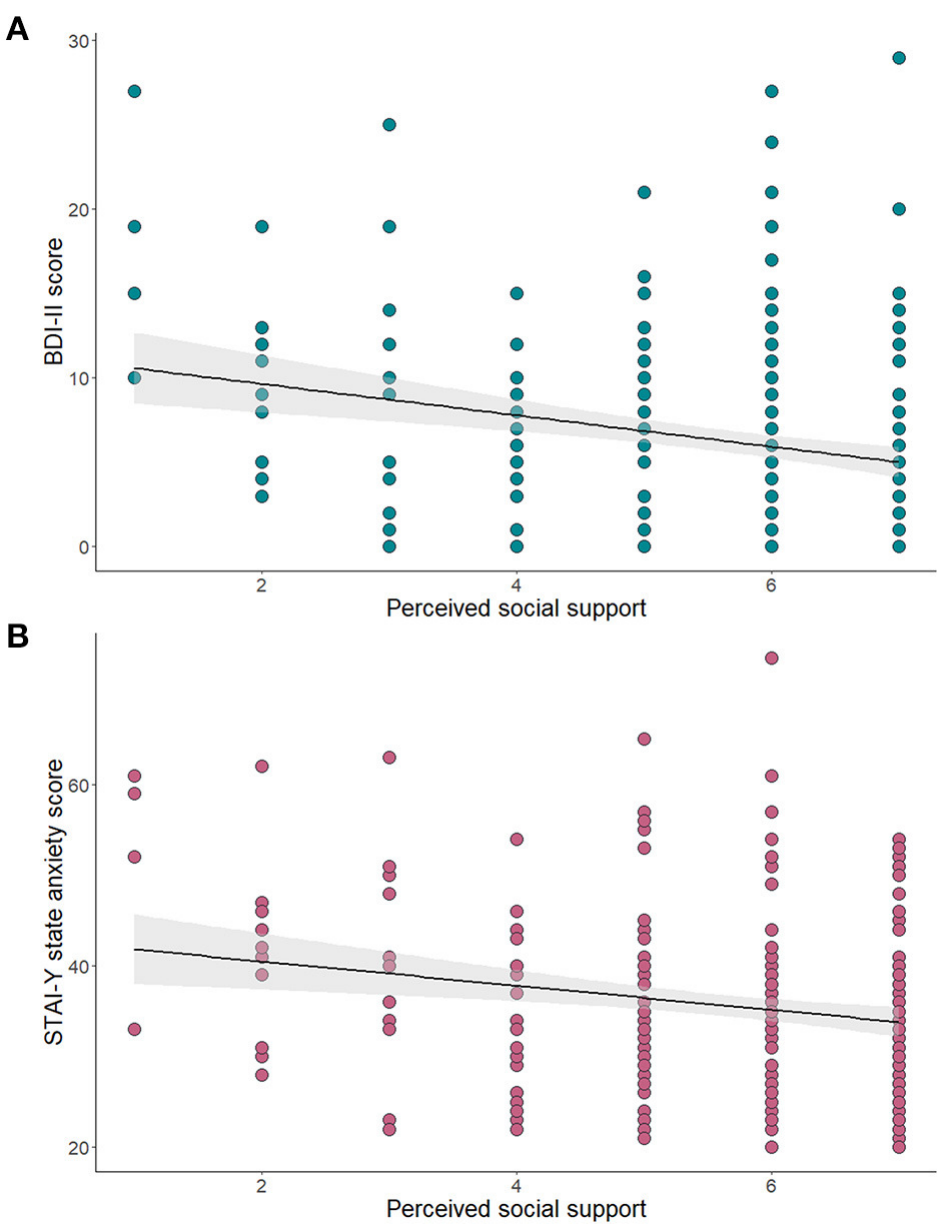
Association of perceived social support during pregnancy and continuous scores of symptoms of depression **(A)** and anxiety **(B)**.

Perceived social support during pregnancy was significantly and negatively correlated with both symptoms of depression, *r* = −0.25, *p* < 0.001, and anxiety *r* = −0.21, *p* = 0.001 (Figure 3). One unit increase in perceived social support was significantly associated with a lower risk of developing clinically significant anxious, B = −0.31, *p* = 0.001, Exp(B) = 0.73, 95% C.I. (0.61:88), and depression, B = −0.36, *p* < 0.001, Exp(B) = 0.70, 95% C.I. (0.58:0.84).

## Discussion

The present study reports on the mental health of women who were pregnant and gave birth to their infants during the 2020 COVID-19 outbreak in northern Italy. The first specific aim of the study was to assess the presence of significant differences in symptoms of depression and anxiety among mothers exposed or not exposed to the SARS-CoV-2 virus. In contrast to our initial hypothesis, there were no statistically significant differences in affective symptoms self-reported by mothers who had at least one direct or indirect exposure to COVID-19 and those who disclosed no exposures. In other words, being themselves positive for COVID-19 or having relatives or friends who were hospitalized or died with COVID-19 infection were not factors associated with significant symptoms of depression and anxiety. Even in the absence of direct or indirect exposure to the SARS-CoV-2 virus, one-quarter of the sample reported clinically relevant depressive symptoms and approximately one-third reported clinically relevant anxious symptoms in the immediate post-partum. Previous reviews and meta-analysis about the pre-pandemic period estimated that postnatal depression affected approximately 17% of all women (45), and about 12% of healthy mothers without previous depressive episodes develop a full-blown post-partum depression (46). The pre-pandemic prevalence rate of post-natal anxiety symptoms was 15%, while the rate of full-blown anxiety disorders was about 10% (12). In our sample, the high percentage of mothers reporting clinically significant depression (26%) and anxiety (32%) suggests an increase in post-natal affective symptoms' rates during the pandemic. Therefore, women who gave birth to their infant during the COVID-19 emergency may be facing a relevant emotional and psychological burden and they should be considered a potentially vulnerable population that may require psychological support.

The second specific aim was to assess the association between pandemic-related prenatal emotional stress and both post-natal depressive and anxious symptoms. Results showed that the extent of self-reported emotional stress response to COVID-19 emergency was significantly associated with a higher risk of both depressive and anxious symptoms. This association was reported both with the continuous score for BDI-II and STAI-Y as well as with the dichotomous risk score for clinically relevant symptomatology. The present findings are consistent with previous literature showing that prenatal stress may increase maternal depressive and anxious symptoms in the postpartum period (12–17). Moreover, these findings suggest that not only the emotional stress experienced by pregnant women during the COVID-19 emergency may associate with transient and subthreshold affective symptoms; rather, it may dramatically raise the risk of full-blown depression and anxiety. These findings are further concordant with previous similar reports on postnatal maternal mental health during the present healthcare emergency from Italy (47) and other countries (24, 48–50).

The third specific aim of the present study was to investigate the association between perceived social support during pregnancy and postnatal symptoms of depression and anxiety. In line with the hypothesis, results showed that the social support perceived by mothers was significantly associated with a reduction in the severity of anxious and depressive symptoms and with a lower risk of developing clinically relevant affective problems. Consistent with previous literature, the availability of social support represents a protective factor that may result in a reduced risk of adverse psychological conditions after delivery (33–35, 37). Notably, the odd ratio linked with the buffering effect of social support was far lower than the one for the association of emotional stress with depressive and anxious symptomatology. This finding suggests that the availability of family support may be only a partial resource for pregnant women during a global healthcare emergency; moreover, mitigation and containment strategies have further reduced the level of social support on which they could rely. In this scenario, timely preventive actions that may provide families with adequate access to psychosocial support should be prioritized to favor the mental health of pregnant women, even during a healthcare emergency.

Previous research documented that low maternal mental health during the first months of life may have detrimental effects on infants' development (51). For example, maternal prenatal depression may have programming effects on infants' temperament and behavioral regulation through neuroendocrine pathways and inflammatory cytokines (52). Moreover, prenatal anxiety may not only associate with infants' socio-emotional outcomes (53), but it may also contribute to less-than-optimal cognitive development (54). As such, promoting maternal healthcare during the COVID-19 pandemic should be considered not only beneficial for women's health but also a preventive intervention for infants' well-being and development.

Finally, it should be highlighted that only healthy mothers and infants were enrolled in the present study. In the light of these results, it is, therefore, possible that the psychological impact of the COVID-19 emergency might be even more relevant for parents of at-risk infants (e.g., preterm birth, perinatal morbidities). For instance, the restriction to parental visiting in neonatal intensive care units may exacerbate the stress experienced by parents during this unprecedented healthcare emergency (55).

## Limitations

First, data were only collected using self-report tools, and those focused on COVID-related variables (exposure and emotional stress) were developed for this study. Nonetheless, these tools showed adequate internal consistency. Second, the study is cross-sectional and data for what pertains to mothers' depressive and anxious symptomatology before the COVID-19 emergency and during pregnancy were not collected. Third, symptoms of depression and anxiety were assessed within 48 h after the childbirth, a period during which transient affective difficulties (e.g., low mood, irritability, and sadness) are quite common and typical of the well-known maternity baby blues. Nonetheless, compared to the pre-pandemic prevalence rates of post-natal affective difficulties our results suggested an increase in post-natal affective symptoms, regardless of whether they are transient or may evolve into full-blown affective disorders. Fourth, all the enrolled subjects lived in northern Italy and the findings may be partially extended to other populations in absence of replications.

## Conclusion

Women who became mothers during the COVID-19 emergency appear to be at high risk for developing mental health problems (i.e., higher risk of anxiety and depressive symptomatology) due to emotional stress and partial social support. Further longitudinal research is needed to assess the development of maternal affective problems during the post-partum period and their potential effects on the infant. Moreover, potential psychological and biological moderators and mediators should be investigated (56–58). The promotion of maternal mental health should be pursued and promoted during and after the COVID-19 pandemic (59) and may serve the double scope of supporting maternal mental health and preventing detrimental consequences for the growth and development of infants during the first year of life (10).

## Data Availability Statement

The raw data supporting the conclusions of this article will be made available by the authors, upon reasonable request.

## Ethics Statement

The studies involving human participants were reviewed and approved by Ethics Committee, University of Pavia. The patients/participants provided their written informed consent to participate in this study.

## Author Contributions

SG and LP: study conception, methodology, data analysis, and draft. PA, GB, AC, LD, RF, EF, BG, RGia, PG, EG, MM, EM, RN, DP, CP, FP, CS, BS, MS, and AS: data collection and data management. RGio, SO, and RB: scientific advisory, supervision, and methodology. All authors contributed to the article and approved the submitted version.

## MOM-COPE Study Group

Monica Albini, Giulia Bensi, Elisa Bettiga, Renza Bonini, Rosanna Bucci, Giovanna Centinaio, Giuliana Del Campo, Alessia Di Marco, Andrea Gitti, Paola Guerini, Gaia Kullman, Maria Roberta Longo, Laura Malerba, Silvia Malguzzi, Paola Martelli, Mario Motta, Cristiana Pavesi, Astrid Pedranzini, Benedetta Chiara Pietra, Pierangelo Veggiotti, Luisa Ventura, Patrizia Vergani, Sonia Zatti, Marco Zecca.

## Conflict of Interest

The authors declare that the research was conducted in the absence of any commercial or financial relationships that could be construed as a potential conflict of interest.

## Publisher's Note

All claims expressed in this article are solely those of the authors and do not necessarily represent those of their affiliated organizations, or those of the publisher, the editors and the reviewers. Any product that may be evaluated in this article, or claim that may be made by its manufacturer, is not guaranteed or endorsed by the publisher.

## Footnotes

^1^Data from the Italian Ministry of Health observatory, last updated July 9th 2021, https://www.salute.gov.it/portale/nuovocoronavirus/dettaglioContenutiNuovoCoronavirus.jsp?id=5367&area=nuovoCoronavirus&menu=vuoto.
